# Does gynaecological cancer care meet the needs of Indigenous Australian women? Qualitative interviews with patients and care providers

**DOI:** 10.1186/s12913-019-4455-9

**Published:** 2019-08-29

**Authors:** Beverley Marcusson-Rababi, Kate Anderson, Lisa J. Whop, Tamara Butler, Nicole Whitson, Gail Garvey

**Affiliations:** Charles Darwin University, Wellbeing and Preventable Chronic Diseases Division, Menzies School of Health Research, Casuarina, NT 0811 Australia

**Keywords:** Cancer, Indigenous Australians, Gynaecological cancer, Patient-centred care, Cultural safety

## Abstract

**Background:**

There is a disparity in the burden of gynaecological cancer for Indigenous women compared with non-Indigenous women in Australia. Understanding how Indigenous women currently experience gynaecological cancer care services and factors that impact on their engagement with care is critical. This study explored Indigenous Australian women’s experience of gynaecological cancer care at a major metropolitan hospital in Queensland.

**Methods:**

Indigenous women receiving care at a major metropolitan Queensland hospital for investigation or diagnosis of gynaecological cancer were invited to participate in a larger longitudinal study exploring women’s experiences of gynaecological cancer care. This component was an in-depth, qualitative interview exploring the women’s experiences of hospital care at approximately three-month post initial referral. A peer-approach was used to interview women. Hospital-based care providers involved in the care of Indigenous gynaecological cancer patients were invited to be interviewed. Interviews were transcribed and thematically analysed using an interpretative phenomenological approach enabling a multi-layered, contextualised understanding of the patients' experience and their interaction with tertiary cancer services.

**Results:**

Eight Indigenous patients and 18 care providers were interviewed. Analysis of all interviews revealed four broad issues affecting Indigenous patients’ early experiences of care: (1) *navigating the system*, impacted by timely diagnosis, access to support services and follow up; (2) *communication and decision-making*, patients’ decision-making, efficacy of doctor-patient communication, and patients’ knowledge about cancer; (3) *coping with treatment demands*, was impacted by emotional stress, access to services and support by hospital staff; and (4) *feeling welcome and safe in the hospital*, impacted by patients’ relationship with care providers and their access to culturally-safe services. The combination of factors impacting these women’s’ experience of gynaecological care commonly left these women at breaking point, often with limited access to information, resources or support.

**Conclusions:**

Our findings revealed that experiences of cancer care for Indigenous women are overlain by challenges associated with late referral, misdiagnosis, miscommunication, lack of information, logistics in accessing treatment and services and system cultural insensitivities. Our findings offer insights that can inform cancer care provision to more effectively support Indigenous women accessing gynaecological cancer services.

**Electronic supplementary material:**

The online version of this article (10.1186/s12913-019-4455-9) contains supplementary material, which is available to authorized users.

## Background

Cancer is a leading driver in the health gap between Aboriginal and Torres Strait Islander people (hereafter respectfully referred to as Indigenous Australians) and non-Indigenous people residing in Queensland, Australia [1–3]. While the overall cancer incidence rate for Indigenous Queenslanders is slightly lower than for non-Indigenous Queenslanders (464.1 vs. 471.0 per 100,000 respectively), the age standardised incidence rate of gynaecological cancers is markedly higher for Indigenous women [4], which aligns with the national data for gynaecological cancers in Aboriginal and Torres Strait Islander women [5]. The term *gynaecological cancer* refers to a group of cancers that occur in the female reproductive area and include cervical, ovarian, uterine, vaginal, vulva, fallopian tube and placenta cancers [6, 7]. Most notable among these cancers are the substantially higher incidence rates for uterine (40.9 vs. 17.6), ovarian (16.6 vs. 10.6) and cervical (15.2 vs. 7.6) cancers, for Indigenous women compared to their non-Indigenous Queensland counterparts [4].

The cancer mortality rates for Indigenous women are higher than for non-Indigenous women in Queensland; for example, uterine (3.6 vs. 3.2 per 100,000, respectively), ovarian (7.8 vs. 6.4) and cervical cancer (4.6 vs. 1.9) [4]. Gynaecological cancer specific survival differences are lower for Indigenous women compared to non-Indigenous women, particularly in the first year following diagnosis [8].

The contributing factors for this disproportionate burden for Indigenous women diagnosed with a gynaecological cancer, include: being diagnosed with more advanced disease; having a higher burden of comorbidities; receiving less cancer treatment overall; and less optimal treatment [8]. However, the factors driving these disparities are complex and cumulatively affect Indigenous people in their readiness and ability to engage with cancer care services. Existing qualitative research involving Indigenous cancer patients and cancer health professionals has identified several factors impacting engagement, including: contextual factors moderating trust in the health system [9, 10]; access to health services [9, 11–14]; communication and decision-making [10, 11, 15]; cultural-safety of health services [9–11, 15]; and individual and community views about cancer [9, 11]. Another study involving a small sample of Aboriginal women with gynaecological cancer and clinicians found that patients had negative experiences involving timely access to health services; access to culturally appropriate screening; family and familiar Indigenous staff; gender of health professionals; and the provision of appropriate educational materials and explanations [16]. Little is known about whether, and to what extent, these or other factors impact engagement with cancer services and cancer outcomes for Indigenous women with gynaecological cancers in Queensland.

Cancer Australia’s National Aboriginal and Torres Strait Islander Cancer Framework states that good quality cancer care for Indigenous Australians must be ‘person centred so that the whole person (including the family and cultural role) is considered, and the psychosocial, cultural and supportive care needs and preferences of Aboriginal and Torres Strait Islander people are addressed across the continuum of care’ [17]. Positive patient experiences have been reported to be associated with improved clinical effectiveness and patient’s safety [18].

To determine what constitutes culturally-safe patient-centred care for Indigenous women with gynaecological cancer in Queensland, it is critical to understand how this patient group experiences cancer care services. Qualitative research that explores multiple perspectives of this type of complex phenomenon (i.e., patient and care provider perspectives) is thought to be the most appropriate method for ensuring comprehensiveness of views and encouraging reflexive analysis of the data [19], while also utilising research methods that are acceptable and familiar to Indigenous people [20].

A mixed methods study was conducted to explore the experiences of gynaecological cancer care by Indigenous women at one hospital site in Queensland in 2016–2017. This paper reports on the qualitative component of the study that investigated the perspectives of Indigenous women and cancer care providers (CCPs) from a large tertiary hospital in Queensland to understand the nature of current cancer care provision and how it is experienced by this patient group. Patients’ views on the gaps in current cancer care offerings and factors that impact on their engagement with care are reported in this paper. The inclusion of both patient and CCP views in this study facilitated a contextualised understanding of how gynaecological cancer care is currently experienced by Indigenous women and how this care provision could be adapted to better meet the needs of this patient group.

## Methods

### Participants and data collection

The study population is as follows.

- Indigenous women aged 18 years or over, who were referred to the Gynaecological Oncology Department of a large tertiary hospital in Queensland for gynaecological cancer care – including diagnostic testing and treatment – between September 2016 and July 2017. Women were invited to participate if they had a diagnosis of, or were suspected of having, gynaecological cancer and were currently attending the Hospital’s Gynaecological Oncology Department for investigation and/or treatment. Those who were not physically and/or mentally well enough, as determined by the clinical nurse coordinator, were excluded from the study.
- CCPs involved in the care of Indigenous gynaecological cancer patients at the hospital site.

Participants were purposively sampled based on the inclusion criteria described above. Women who fit the inclusion criteria were initially approached about the study by phone by a hospital-based member of the research team (clinical nurse coordinator from the Gynaecological Oncology Department).

Indigenous women (patients) were invited to participate when the nurse contacted them to confirm their hospital appointment with the Gynaecological Oncology Department. During this phone call potential participants were informed about the study and asked if they were interested in participating. Those who agreed gave consent to have their contact details passed onto one of two experienced Indigenous researchers (BM, NW). The interviewer subsequently contacted the patient to arrange a convenient time to conduct the interview.

CCPs were purposively sampled and were interviewed once. CCPs included a range of professional roles, including: clinical, allied health, administration and support roles.

The interviews were guided by semi-structured interview guides (see Additional file 1: Interview Guides for Patients and Health Professionals), which provided an outline of topics to be discussed but also allowed participants the opportunity to describe their experiences in their own words. All interviews were conducted in English. The interview guides were developed and informed from a literature review and input from the research team. The patient interviews focussed on their experiences of cancer care relating to the topics of: communication with health staff; decision-making processes; receiving treatment; barriers and enablers to accessing services; support needs; and their overall experiences of cancer care. The CCP interviews focused on their professional roles and experiences with Indigenous women with gynaecological cancer relating to the topics of: communicating with patients; informing patients about their cancer and treatment; their views on barriers and enablers to Indigenous women accessing and engaging with cancer care; and general reflections on providing care to this patient group.

Socio-demographic information was collected from the patients via self-report, including: age, Indigeneity, sex, marital status, employment, educational level, and main language spoken at home. Clinical information was extracted from the patients’ medical records by a hospital-based member of the research team from medical charts using a standardised template. Charts were reviewed approximately 6–9 months after the initial interview to collect information regarding the participants’ cancer diagnosis, date of diagnosis, gynaecological cancer type, and comorbidities (presence/ absence of diabetes, hypertension, cardiovascular disease, renal disease and/or respiratory disease). Information collected from the CCPs included: sex, Indigeneity, and how long they had worked in their current roles at the participating hospital site.

The qualitative interviews were conducted with participants (patients and CCPs) between February 2017 and October 2017 by one or two of three experienced researchers (BM, NW, KA); none of whom had any affiliation with the hospital or the patients. At the time of interviewing, KA had over 10 years’ and BM had over 5 years’ experience in Indigenous health research, and NW was in the final year of studying Medicine. At least one Indigenous researcher was in attendance at all interviews. This use of peer interviewing is important when conducting research with vulnerable and hard-to-reach populations to ensure that the participants are comfortable in telling their story without fear of negatively impacting their medical care [21]. All interviews were audio-recorded with the participants’ consent.

### Data analysis

All interviews were transcribed verbatim, de-identified and imported into NVivo11 software (QSR International Pty Ltd., Doncaster, Victoria) for analysis. The duration of the interviews was between 25 and 60 min. Two researchers (BM and KA) thematically coded the manuscripts line-by-line. Inductive and descriptive analyses were guided by an interpretive phenomenological approach that enables insights into the lived experience of interviewees [22]. Emerging themes were identified and negotiated iteratively between the two researchers (BM and KA), until a final thematic framework was determined. The results presented here make use of this thematic framework. The inclusion of both patient and CCP views facilitated a triangulation of perspectives, allowing a contextualised understanding of how care is both provided for and experienced by Indigenous women. The responses of patients and CCPs were analysed and reported separately, with differing perspectives between the two groups being identified and explored.

The sociodemographic and clinical characteristics of the eight Indigenous patients included in this study were described using proportions (%) for categorical variables and range and average to describe the age of the women at time of interview. Due to small participant numbers, the categories were often collapsed and dichotomised. Socioeconomic Index for Areas (SEIFA) Index of Relative Socioeconomic Advantage and Disadvantage (IRSAD) [23] was used to classify patients into deciles from most to least advantaged, according to their residential postal codes. The deciles were then collapsed into least advantaged (decile 1–5) and most advantaged (decile 6 to 10). Postcode was also used to assign an Accessibility/Remoteness Index of Australia (ARIA+ 2011) [24] score to each woman, which was then categorised into highly accessible (e.g. *major* cities) and all other areas.

## Results

A total of 26 participants (eight Indigenous patients and 18 CCPs) were included in this study.

Eight patients participated in a qualitative interview. All were Indigenous and most identified as Aboriginal (*n* = 5). The women were aged between 33 and 68 years at the time of interview, with an average age of 52 years. At the time of the interview, half of the patients reported their marital status as single (*n* = 4). Most patients reported having children (*n* = 7), and all (*n* = 8) spoke English as their main language at home and were not in paid employment. The reported education level of patients was evenly split between those having year 10 or below (junior) high school education (*n* = 4) and those who achieved year 12 (senior) high school or vocational education training (*n* = 4). Most patients came from geographical areas that were classified as being highly accessible (*n* = 5), but in the least advantaged areas (*n* = 7). Two of the eight Indigenous patients were accompanied by a support person (family member or Indigenous Liaison Officer). This person occasionally assisted the patient during the interview by re-phrasing the question or assisting the patient to think through their response.

The women were diagnosed with a range of gynaecological cancers including: endometrial cancer, ovarian cancer, cervical cancer, and vulva cancer. One woman was suspected of having uterine cancer, but was later cleared. Seven of the women had chronic comorbidities, including diabetes, hypertension, cardiovascular disease, and respiratory disease. Two women had a previous cancer, three were obese, two had depression and two had other gynaecological conditions. Four of the women had undergone surgery for their gynaecological cancer, with another woman who had surgery planned. Two of the women had undergone chemotherapy and none had undergone radiotherapy. One woman passed away before the end of the study period.

The professional roles of the CCPs were varied and included: senior surgical registrar (*n* = 1); medical oncologist consultant (*n* = 1); clinical nurse consultant (*n* = 1); senior social worker (*n* = 1); Indigenous Liaison Officer (*n* = 2); physiotherapist (*n* = 2); pharmacist (*n* = 3); pastoral care worker (*n* = 3); and oncology nurses (*n* = 4). Of the staff, 15 were female (83%); three Indigenous (17%); and 78% (*n* = 12) of them had worked in their current roles for 4 years or longer.

**Fig. 1:**
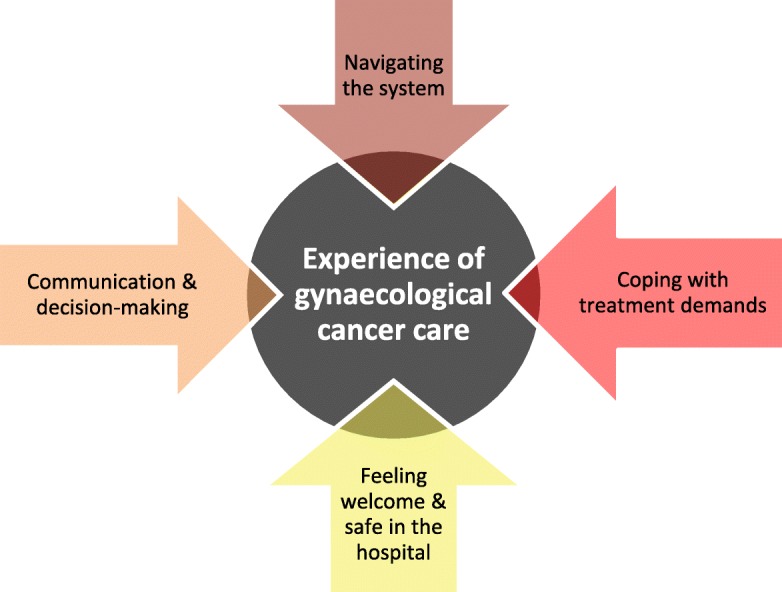
Issues impacting Indigenous women’s experience of gynaecological cancer care

Analysis of the patient and CCP interviews revealed four broad issues that greatly impacted Indigenous patients’ experience of gynaecological cancer care: navigating the system; communication and decision-making; coping with treatment demands; and feeling welcome and safe in the hospital (Fig. 1).

### Navigating the system

#### Receiving a timely diagnosis

Patients and CCPs identified late diagnosis as an issue impacting the experiences of cancer care for many Indigenous women. Those patients who reported being diagnosed at a late stage of cancer described their experiences of care as highly stressful, frightening and rushed. Due to the urgency to commence treatment, these patients felt they had little understanding of their cancer and the treatment, and were dismayed at their poorer health prospects due to their late diagnosis. CCPs also commonly reported that Indigenous women commonly present for treatment at a later stage of cancer; which limits available treatment options.

*Finding out you’ve got cancer is a big shock to begin with, but when I went to the [Hospital], I didn’t find it too bad there, but they didn’t give me any information when I left to know what was going to happen to me after. About a month ago I went into anxiety mode and went to the doctor’s crying my eyes out for hours. – PT, 15*

CCPs predominately attributed late diagnosis of Indigenous women to their delayed presentation due to reduced access to services, particularly in remote areas, and their limited knowledge of cancer symptoms. Additionally, CCPs commonly described Indigenous women as *stoic*. This term was used to explain delayed diagnosis as Indigenous women were said to typically endure symptoms and pain far longer than non-Indigenous women. In contrast, some patients described experiencing significant delays in being referred by their GP to a cancer specialist which resulted in long delays before receiving a cancer diagnosis and subsequent treatment. Patients attributed the delay in being diagnosed with cancer to: long wait times for referral to and appointment with a cancer specialist; geographical isolation from services, and GPs’ lack of awareness in the presentation of gynaecological cancers. These delays were a source of frustration and anger for these patients.

*It was like a big rollercoaster because I was rushed through. Within a month, I was having [surgery]. I went to all different doctors and everything and it was just so fast. It was just like a rollercoaster ride because they knew if they left it any longer; it would be too late. . . I was angry at the doctor because I had told him that I needed a biopsy and he said that I didn’t need one . . . People are dying because they are waiting eight months to go in to get a cancer looked at . . . The gynaecologist only had to take one look and knew it was cancer. So, why didn’t the [GP] know when he took a look at it? That’s what I want to know. Aren’t they educated in that? – PT, 14*

#### Access to services

Issues related to distance and remoteness from the hospital service impacted the experiences of several patients and was a common issue identified by many CCPs. The participating hospital’s large catchment area was said to particularly affect Indigenous patients, as many commonly travelled from regional and remote communities to access this hospital-based gynaecological oncology cancer service. Funding cuts and changes of government were described as producing instability in program and service delivery to regional and remote Australians. This was described as a significant structural barrier facing Indigenous women accessing cancer services, as well as undermining confidence and trust in these services by Indigenous women due to the inconsistency of cancer programs in regional and remote areas. Travel to and from the hospital also posed difficulties for patients living in outer regional areas. One woman described her anguish after receiving little assistance from hospital staff when she was sent home on a long public bus trip after surgery with significant vaginal bleeding and discomfort.

*I went in on the bus on my own and I come back on the bus on my own, but I hadn’t been sitting yet …one of the nurses said to me… “I know you haven’t sat yet . . . I’m going to put a couple of towels in your bag” . . . Now, when you get on the bus,” she said, “just roll these up and sit on them.” PT, 14*

#### Support and follow-up

Several patients reported that attending hospital appointments is difficult when you are ill. They described feeling that care providers interpret non-attendance at appointments as the patient not caring and label them as ‘non-compliant’. Patients described feeling abandoned by the health system and wanted greater support and more flexibility from care providers to maintain their connection to cancer services. Patients reported wanting more contact and follow-up care after treatment. Without sufficient follow-up, patients felt confused and anxious about their treatment plan and prognosis.

*… I just don’t feel that I can go to the appointment or whatever, it’s like they think, okay, she’s not even looking after herself, so why bother. My doctor said he’s coming to see me, he didn’t. Nothing has been done. I just feel like I’m left here and they don’t care. - PT, 16*

CCPs talked about a perceived reluctance among Indigenous patients to attend appointments, particularly when patients are asymptomatic. CCPs ascribed this reluctance due to financial and logistical barriers and mistrust of the medical system. Additional reminders and phone calls with Indigenous patients were described by some CCPs as a mechanism to encourage them to attend hospital appointments. Some CCPs said that while providers often say that Indigenous patients are ‘non-compliant’ with their cancer treatment, they really ‘*have no idea of what these women are having to deal with when they go home, or back to their communities’* – *CCP, 18.*

The assistance provided by the Indigenous Liaison Officers (ILOs) to navigate Indigenous patients through cancer care and the health system at the hospital was a notable source of support for several patients. Patients who were introduced to an ILO reported a more positive experience in the hospital than those who were not. It was unclear why some women were not put in contact with an ILO; however, many CCPs reported that the ILO staff had a sizeable workload, which may explain the limited access.

### Communication and decision-making

#### Effective communication and access to information

Communication difficulties significantly impacted the experience of cancer care for many Indigenous patients. The difficulties described by patients included: unsympathetic delivery of bad news, confusing terminology, time pressured appointments, language difference between patients and CCPs, a lack of available interpreters, confusion over written documents, and poor cultural competency in the hospital.

Patients described a lack of sufficient and appropriate information about their treatment plans, follow-up, appointments, side-effects, and availability of support. This left many patients feeling bewildered and stressed.

Some patients also felt that CCPs talked down to them. CCPs’ use of medical jargon often left patients feeling confused, frustrated, and ill-equipped to make decisions about their treatment, with little opportunity to ask questions. Patients reported that the reasons for missed appointments and disengagement with care were feelings of confusion and anxiety relating to notifications and timing of appointments and follow up.

*They were using medical talk and I didn’t understand . . . You see different doctors, so you’re telling your story over and over to different doctors . . . Yeah, got to put up with rude doctors, rude nurses constantly . . . Oh, it just made me angry. I just don’t feel like going to see doctors any more. – PT, 22*

The few patients who reported receiving timely and effective communication about their cancer and treatment reported a more positive experience of care. While some booklets were given to patients, only a few patients described these resources as helpful.

*I had an appointment with the Chemo doctor and yeah, she told me about it. Gave me booklets, and pamphlets and stuff. But yeah, it still didn’t make sense to me… I still didn’t know what I’m going to go through. – PT, 20*

Health professionals reported mixed views on the efficacy of current communication with Indigenous patients. CCPs reported language difference and access to interpreters as common obstacles to effective communication. Patients’ use of escorts or carers as interpreters was seen as ethically problematic; however, CCPs often relied on them, given the limited access to interpreters, particularly for creole speakers. CCPs also described many Indigenous women as shy and not willing to speak up with questions.

*Sometimes we have to use family members which isn’t ideal because, again, it’s all the understanding and relaying - you’ve no idea of what’s actually been said to the patients in terms of emphasis. – CCP, 01*

The ILOs reported that they often undertake informal mediatory roles to facilitate communication between CCPs and Indigenous patients.

*Sometimes you can sit in on the appointment, and you know that the patient has not understood. And usually I’ll go in before … and talk, “The doctor is going to come, he is going to ask you questions about this, this, this and this. If you don’t understand or you want me to say something, you can just look at me, and I can say it, or I can ask the questions… we might sit down and write down a few questions…I’ll sit in there with her when the doctor is talking to her. And you can tell by body language if they don’t understand. So I might say something, usually I’ll go like, “Doctor, just for my understanding, do you mean?” – CCP, 03*

#### Knowledge of cancer and treatments

While many patients had friends and relatives who had experienced cancer, they felt they had limited knowledge about cancer treatment. Many described not only experiencing physical side-effects from the treatment, but also a loss of autonomy and exclusion from decision-making processes. This was particularly so for those patients who were diagnosed at later stages of cancer.

*I was told what was about to happen, I don’t have any input, I can’t say yes, I want or no, I don’t, because they do what they like. – PT, 16*

*You just feel like you’re one of these hundreds of cattle that are going through this bloody thing, you know, a number, but anyway, that’s, you know, you just go it, whatever they say you do. – PT, 14*

Indigenous patients’ limited knowledge of cancer and treatments was put forward by some CCPs as a barrier to women engaging with treatment. CCPs reported that there were only text-heavy educational resources about cancer available, and limited resources tailored specifically for Indigenous patients.

*Once they get down here I think they would be given exactly the same information, but whether that suits their specific requirements I don’t know. – CCP, 01*

### Coping with treatment demands

#### Emotional stress and support

The emotional strain of cancer diagnosis and treatment was unsurprisingly evident in the patient interviews. Feelings of shock, fear, anger, shame and isolation were interwoven throughout the patients’ accounts. The strain was commonly heightened due to the issues already identified relating to poor communication, late diagnosis and uncertainty and delays around their treatment and prognosis. A lack of financial support, social work services and accommodation exacerbated stress and anxiety for patients, their family and escorts.

*I’ve got no life; I’ve got nothing to look forward to, except misery … I’m at the stage where I’m sick of medication, I’m sick of doctors, and I don’t really want to have anything to do with them. – PT, 16*

Patients also described that they were commonly dealing with crises overlaying their cancer, including other seriously ill family members, housing insecurity and severe financial stress. The perceived lack of support from hospital staff was too much for several patients to cope with.

*I’m tired of asking. I don’t want it; I don’t want the help, because there isn’t any. - PT, 16*

While CCPs commonly interpreted Indigenous women’s behaviour as lacking concern about their cancer and treatment, patients said that they were experiencing crisis. One patient described that her way of coping with stressful situations is by joking and laughing to mask her distress, which she said was misinterpreted by her clinician as real happiness.

*But I do a happy thing. I don’t show anyone I’m upset … And even when I got in there, he [the doctor] remembered me, because he’s never seen anyone go through what I’ve gone through laughing. He reckons that I was the happiest patient he’s ever had, but only if he knew the real reason for that, but anyway. I’m always like that, that’s just a normal thing, a reaction of mine that I take on to get over everything. – PT, 14*

#### Logistical challenges

Travelling to a metropolitan hospital is often required for access to diagnostic tests, treatment and cancer services, which presents a variety of logistical difficulties for patients. These difficulties impact on patients from remote and rural areas but also those from urban areas around Brisbane; including challenges associated with transport, finance and accommodation. While remote patients were often assisted with the costs and organisation of travel and accommodation; this was not the case for the local Indigenous patients. The cost of travel, parking and other out-of-pocket expenses meant that many of the patients were under significant financial stress. When patients could apply for reimbursement of expenses, they described an onerous process of filling out and submitting forms, which took a great deal of time, knowledge and effort to complete. Patients often required the assistance of Aboriginal Community Controlled Health Organisations to complete these forms.

*With paying for hospital and whatnot, we have put the car off the road, to get me here I guess, into the hospital – PT, 11*

Patients described difficulties accessing ‘Closing the Gap’ Pharmaceutical Benefits Scheme (PBS) Co-payment scheme in hospital and found they were commonly out-of-pocket for medications and other treatment related expenses.

*I got [Closing the Gap Co-payments] through my doctor, but whenever I said something to them at the hospital they kind of looked at me as if I was crazy. – PT, 11*

Some patients who relocated for treatment from remote and regional communities experienced increased anxiety and distress caused by the move, as some had never left their community before. Patients described the experience of the air travel and being in a big city, while dealing with a new cancer diagnosis, as shocking. These patients felt the loss of social support from family and community keenly. Housing was also a major issue for patients who had to relocate for extended periods to receive their cancer treatment. A lack of appropriate and accessible accommodation raised issues for several patients, which resulted in additional stress on top of dealing with their cancer diagnosis and treatment.

*I’ve got to have my carer with me, and it’s going to be difficult to find somewhere, but I’ve got so many other problems on top of me as well, that it’s just, I don’t know where to start. – PT, 16*

Some CCPs reported awareness of difficulties relating to accommodation, transport and financial stress facing many Indigenous patients. However, other than putting these patients in touch with the hospital ILOs or social workers, CCPs did not view these issues as their responsibility.

*My experiences are that the people, especially travelling long distances, are anxious, they’re completely out of their element, they don't know what’s going on, they’ve never been to big cities before and it's completely overwhelming, and the more services you can get and the more contact you can have with Aboriginal Liaison services or get them that support, it makes their journey a lot easier. CCP, 01*

#### Support from hospital staff

Only a small proportion of patients in this study reported seeing a social worker. There was confusion among patients about where to access support and what sort of services and resources were available to them. A couple of patients reported positive experiences with social work; one woman received some financial support and another received a teddy bear for her children. Given the substantial support needs of this population, the access to social work was minimal. This gap was somewhat filled by the ILO services and patients reported positive experiences when negotiating the system with the support of an ILO. However, with only three part-time ILOs working across the whole hospital, access was limited for patients needing intensive support.

Several patients reported being advised by their treating clinicians that they could not undergo required cancer surgery until pre-existing conditions of obesity and overweight were addressed. CCPs talked about the value of allied health services, such as dietitians and psychologists, in assisting patients to lose weight prior to surgery. However, patients’ experience of these services was often challenging, with appointments only being offered at night, or a perceived a lack of empathy and understanding from staff at these services, causing distress and disengagement with services.

*They said they’d put dietitians and nutritionists to talk to me and psychiatrists and psychologists, but talking to me isn’t working, it isn’t doing anything. It’s frustrating me, and it makes me angry. PT, 16*

### Feeling safe and welcome in the hospital

#### Relationship with CCPs

Patients reported mixed feelings towards their relationships with CCPs and the quality of these relationships emerged as an important moderator of their experience of cancer care. Issues of trust, rapport, cultural sensitivity and respect were key determinants of patients’ satisfaction with health professionals. These issues impacted on patients’ confidence in the treatment process and their commitment to engaging with cancer services.

*And I said to him [the doctor], “Have you read that I’ve had cancer?” And he said, “Oh, where did you have cancer?” I said, “Down below, where I told you it was.” And he went, “Oh, that’s not good.” And I said, “No, it’s not.” But that’s it. That’s it, that’s all he said. So, I just got up and walked out, and I haven’t been back since. – PT, 14*

Many CCPs were aware of the importance of developing rapport and trust with their Indigenous patients, particularly for patients from remote communities, due to the additional stress that these patients feel in the unfamiliar and often unfriendly hospital environment.

*Establishing rapport is the absolute number one that I would see, and that’s what I see with the compliance. The ones we have the good rapport with, yeah, tend to come in. – HP, 18*

One CCP explained that many health professionals are unaware of the living conditions and cultural contexts of the Indigenous patients they treat. While this was described as not impacting the care provided, these comments suggest that a basic understanding of patients’ backgrounds, values and needs is critical in effectively engaging with Indigenous patients.

*I do know it’s easier for us to know where they come from. Recently someone photocopied exactly where the patient was, right up the top of Australia, and it made us understand more and there was a copy of that on the chart so everyone that came across realised, oh, wow, she’s so far away from home; not that you treat them differently but it’s just more acceptance of what they’re going through being in a place that they’ve never been before. – HP, 02*

#### Cultural sensitivity and consideration

A few patients spoke about feeling culturally alienated in the hospital environment. The disconnection from nature and country while in the hospital caused great unease for some patients. Additionally, patients described feeling stigmatised and ostracised while in the hospital. Posters depicting Indigenous people with hepatitis were the only Indigenous symbols patients reported seeing in the hospital, which caused a sense of shame. One patient felt that she was treated like a drug addict when trying to fill her prescription at the hospital pharmacy, attributing this to racist attitudes toward Indigenous patients.

Some CCPs reported that Indigenous women with gynaecological cancer feel uncomfortable being treated by male doctors, especially patients from coming from traditional communities. However, there were not always female medical staff members available to treat patients. CCPs described ILOs as playing a central role in making Indigenous patients feel culturally-safe and respected. CCPs described support from the ILOs to ‘*walk the journey alongside*’ (*HP, 18*) patients as important in building rapport with Indigenous patients and helpful in encouraging patients to attend appointments. Several patients describe receiving support from an ILO with regards to accommodation, finding their way around the hospital, accessing services and also having a yarn. While the ILOs were seen as invaluable supports by patients, this did not redress the negative impacts of the unwelcoming hospital environment on patients.

## Discussion

This is the first study to explore Indigenous women’s experiences of gynaecological cancer care in Queensland. The findings reveal that Indigenous women accessing gynaecological cancer care are encumbered by additional challenges overlaying the impact of having a diagnosis of cancer. The Indigenous women in the study faced substantial challenges associated with late referral, misdiagnosis, miscommunication, lack of information, logistical challenges in accessing treatment and services, background life crises, and cultural insensitivities in the system. While some of these issues are not in themselves particular to this population, it is the accumulation of these challenges that negatively impact on these women’s experiences of and engagement with cancer care. The overarching picture that emerges through this analysis is of a group of women at breaking point, often with limited access to resources and support. Cancer care services need to account for the commonality of these issues facing Indigenous women with gynaecological cancer and adapt their services and models of care to better support them through their cancer journey.

Shahid and colleagues have reported that system-level change is required to improve coordination between primary and tertiary cancer diagnostic and treatment services, to ensure appropriate levels of social support are provided to Indigenous patients [25]. The challenges facing Indigenous women living in remote locations to engage with and maintain cancer treatments in metropolitan setting were illustrated by both patients and CCPs. Issues identified, such as loss of social support, culture shock, and inappropriate accommodation and interpreter arrangements, echo previous findings from other Australian studies [10, 11, 25, 26]. Logistical challenges also faced patients living closer to the hospital. Financial strain, housing and travel difficulties and family responsibilities all impacted patients’ ability to maintain their engagement with cancer care. Patients strongly expressed greater needs of assistance and support from hospital staff and continuity of care after discharge and treatment [12]. Tools, such as the Supportive Care Needs Assessment Tool for Indigenous Peoples (SCNAT-IP), have been shown to assist care providers in identifying the unmet support needs of Indigenous patients in Queensland [27]. Cancer care coordination has also been shown to significantly ameliorate many problems associated with navigating the health system for Indigenous cancer patients [15]. The issue of paying out-of-pocket expenses associated with cancer care upfront requires attention, as Indigenous people are less likely to have the necessary resources to pay such costs and claim them back later [28].

The increased stress and confusion among patients stemming from communication issues was a recurrent concern, undermining patients’ confidence in health providers and the health system. Indigenous women felt disempowered by the lack of information they received about their cancer diagnosis and treatment, and felt compelled to accept treatments without fully understanding what was involved. Similar patterns have been identified in other studies involving cancer and other chronic diseases [25, 26]. Research suggests that many Indigenous Australians have low awareness and knowledge of cancer prior to a cancer diagnosis [29]. Similarly, Indigenous Australians often inaccurately perceive cancer as a death sentence, which can produce a sense of fatalism and an ensuing avoidance of being diagnosed with cancer [30, 31]. These findings suggest that Indigenous women with gynaecological cancer should be provided with additional information and support throughout the cancer care continuum, particularly at the time of diagnosis. CCPs need to also be aware of cultural differences in communication styles and coping behaviours. The finding in this study of Indigenous women masking their distress through jokes, laughter, and shyness echoes findings in other studies [10] and needs to be considered in all interactions with CCPs.

The stress of dealing with a cancer diagnosis and treatment was often exacerbated for Indigenous women by the compounding logistic challenges and the patients’ living circumstances. The common assumptions held by many CCPs that Indigenous women are stoic and can cope with high levels of discomfort and stress appears to mask the crisis many patients experience. Miscommunication and misunderstanding between the CCPs and Indigenous patients led to many patients feeling unsupported and uninformed, which lead to mistrust and disengagement with health services. Fundamental to receiving optimal care is effective doctor–patient communication. Cass et al. (2002) reported for Aboriginal patients this requires having significant input into the management of their illness and access to educational resources to facilitate a shared understanding of the disease and treatment, but also consideration of the cultural, social and economic aspects of the illness experienced by Aboriginal people [32]. Indigenous women in this study reported feeling that they are often treated without compassion by CCPs, and without regard to their personal and cultural circumstances and values. A high value was placed on the quality of the relationship with CCPs, which has been highlighted by other studies [10, 25, 33]. The critical importance for CCPs to provide patient-centred care to this vulnerable population in order that they feel safe, welcomed, respected and valued, was paramount to fostering a treatment environment where Indigenous women feel encouraged to engage with treatment rather than deterred. A deeper understanding of patients’ circumstances is urgently needed for CCPs in order to adapt their services to suit patients’ needs, values and living circumstances [25]. The value of ILOs is fundamentally important in assisting Indigenous women to feel safe in the system. However, the common reliance on ILOs to support Indigenous patients with all aspects of their interactions with the hospital, shirks the duty of other CCPs to develop their own cultural competence and patient-centred care skills. Instead, ILOs are often overburdened to meet a broad range of needs across many patients. This heavy case load reduces the capacity of ILOs to give sufficient to support to individuals and is a likely reason why many Indigenous patients do not receive a full range of information and support options they need [34].

Our study findings echo and confirm those that Willis et al. [35] identified, highlighting the stark and persistent inadequacies in the provision of culturally-appropriate gynaecological cancer care for Indigenous women. This study further provides new knowledge regarding how Indigenous women experience gynaecological cancer care. In light of these findings, a set of recommendation to improve gynaecological cancer services to better meet the needs of Indigenous women in Queensland has been developed, as detailed below:

### Recommendations for Cancer Care Professionals

- Increase cross-cultural communication training
- Be sensitive to patients’ feelings when communicating diagnosis and prognosis
- Be compassionate and take a whole-of-person approach in your treatment of patients
- Be proactive in offering counselling services to patients
- Be aware of cultural values and preferences when providing cancer care
- Participate in cultural awareness training
- Empower patients to be active decision-makers in their cancer treatment
- Be aware of logistic challenges facing patients in engaging with cancer treatment

### Recommendations for Cancer Care Services

- Ensure patients understand their diagnosis, the nature of treatments and their treatment options
- Produce and make available culturally appropriate patient education resources
- Improve access to Indigenous interpreters
- Make access to allied health support more flexible and convenient for patients
- Employ more Indigenous Liaison Officers
- Employ more Indigenous staff across the service
- Allow escorts and support persons to accompany patients to tests and procedures
- Engage social work services more for Indigenous patients
- Offer more transport assistance for local patients
- Offer more assistance for patients to attend appointments and clinics
- Be aware of socio-economic status of patients and the costs associated with accessing treatment

### System-wide recommendations

- Provide greater training for GPs in cancer identification
- Facilitate more timely referral processes
- Provide patient navigator programs
- Prioritise development of cancer services in regional areas

### Limitations and strengths

This study is limited by the small sample of Indigenous women who were from one hospital site in one jurisdiction. While this may limit the transferability of these findings, the similarity of the current findings with studies in other geographic locations and disease treatment settings suggest their relevance to Indigenous patients in other contexts. The design of this study was also limited by the fact that only the perspectives of those patients who were well enough and willing to participate were included in the sample, which may underestimate the difficulties facing this population. All interviews were conducted by Indigenous interviewers, which may have resulted in some bias in terms of the social desirability of responses. However, this is likely to have improved facilitation of the discussion because of their increased cultural sensitivity towards the issues raised. Despite its limitations, this study has some key strengths. Indigenous women were all from one cancer group and the same service and this may have relevance to cancer care provision in other regions, as well as the chronic disease settings. The inclusion of the CCP perspective enabled the triangulation of perspectives to ensure a comprehensive and reflexive analysis, while employing research methods acceptable to Indigenous people. This study is the first to explore the experiences of Indigenous women undergoing gynaecological cancer care. Given the efficacy of timely treatment for gynaecological cancer, these findings are invaluable for guiding cancer services on how to better support Indigenous women with gynaecological cancer and in turn improve cancer outcomes for these women.

## Conclusions

There is a pressing need for culturally-appropriate, person-centred cancer care and resources to be made available for Indigenous women diagnosed with a gynaecological cancer in Queensland. The experiences of the Indigenous women in this study highlight some critical areas for change to meet their needs and improve the cancer outcomes of this currently underserved patient population. Ongoing engagement with stakeholders – including patients, community members and CCPs – is critical for the development and maintenance of cancer care that will better support Indigenous women in Queensland.

## Additional file

Additional file 1: Semi-structured interview guides. (PDF 471 kb)

## Data Availability

The data collected during this project is not available to researchers outside of the research team due to participant confidentiality.

## Abbreviations

CCP: Cancer Care Provider
ILO: Indigenous Liaison Officer
Indigenous Australians: Aboriginal and Torres Strait Islander People
PT: Patient
