# Supplementary material for: Does gynaecological cancer care meet the needs of Indigenous Australian women? Qualitative interviews with patients and care providers
Source: BMC Health Serv Res. 2019 Aug 29;19:606. doi: 10.1186/s12913-019-4455-9 (PMC6716815; doi:10.1186/s12913-019-4455-9)
Supplement: Supplementary file 1 — Semi-structured interview guides. (PDF 471 kb) [file 12913_2019_4455_MOESM1_ESM.pdf]

Semi-structured interview for patients DIAGNOSED with cancer

The semi-structured interviews will be used to explore Aboriginal and Torres Strait Islander Gynaecological patients' experiences and understanding about their cancer diagnosis, information and treatment choices. As well as their complementary, alternative and traditional medicine use.

Interview guide

Review participant information sheet and participant and interviewer to sign consent form.

Reinforce that participation is voluntary and can be withdrawn at any time. Not all questions have to be answered.

This is your story of your experiences with cancer. There are no right or wrong answers. We are just interested in hearing about your experiences with your cancer, information, treatment and complementary, alternative and traditional medicine use.

We ask the interview to be audio recorded to make sure we accurately represent what you have to say. However, I emphasise that everything you tell me today is completely confidential. Any information that is potentially identifiable will be removed from the interview transcript, and your name will not be recorded anywhere.

**Questions**	
**Treatment to date**	
1	**Tell me about your experience with gynaecological cancer?**
2	**Could you tell me what type/s of cancer treatment you have received at the *[HOSPITAL NAME]*?**<br>*Prompts:*<br>*- Have you had any surgery for the cancer, chemotherapy or radiotherarpy?*
3	**Are you still having ongoing treatment for your cancer?**<br>*If yes,* **What type of treatment?**<br>*If no,* **When did you finish your treatment?**
**Being informed**	
4	**When you found out that you had cancer, did you receive any information about your cancer or your treatment options? [*If yes*] Can you tell me about that?**<br>*Prompts:*<br>*- Can you tell me what type of information did you receive?*<br>*- Did you find that information helpful? How did it help?*<br>*- Pamphlet/flyer - Did you read it? Did you show or talked to your family about it? Was it easy to understand?*
5	**Who explained the treatment options to you?**<br>*Prompts:*<br>*- Could you understand what they told you?*<br>*- Could you tell me what your treatment options are?*
6	**Have there been any other particular difficulties or challenges you have had to deal with since finding out you had cancer?**<br>*Prompts:*<br>*- Can you tell me a bit more about this?*
7	**Do you have any problems understanding what the doctors or nurses explain to you?**<br>*Prompts:*<br>*- Are there interpreters available to you?*

	- Can you ask for more information?<br>*- Is there anything you would like to know more about?*
**Decision making**	
8	*[If patient has received treatment]* **Do you feel like you were given a choice about your treatment? Was this a decision you made together with your doctor or were you happy to follow the recommendation from your doctor?**
9	**Were there other things that you thought about when making the decision about the type of treatment you receive?**<br>*Prompts:*<br>*- Where you live (i.e. geographical remoteness) or your role (e.g., mother/work), time constraints, lack of access to treatment?*
10	**Who did you discuss your options with before making a decision about what treatment to have?**<br>*Prompts:*<br>*- Family members, friends, health professionals*
11	**Did you understand what the treatment involved before you started treatment?**<br>*Prompts:*<br>*- Has any part of your treatment been a surprise to you?*<br>*- How have you coped with the treatment?*<br>*- Are there things that you are worried about? Can you tell me what sort of things?*
12	**What have been the major problems for you following your treatment? Or major benefits?**
13	**What one support (educational, psychological, social support, spiritual, or economic) would have been most helpful to you prior to treatment?**<br>*Follow on questions:*<br>**- And after treatment?**<br>**- When would they have been helpful?**
14	**If you could advise someone who has to make the same decision you did about treatment, would be one thing you would tell them?**
**Experience at *[HOSPITAL NAME]***	
15	**Can you tell me about your experience coming to and whilst at the clinic here at *[HOSPITAL NAME]*?**
16	**Was it easy to get here?**
17	**Have you felt well supported here? If so how and by whom?**
18	**Have you felt respected here? Your beliefs?**
19	**Do the staff members here contact you after your appointments to see how you're going or to remind you when your check-up is due?**
20	**Have you been given information about a plan to feel healthy?**<br>*Prompts:*<br>*- Improving lifestyle choices such as food, alcohol, smoking*

Complementary, alternative and traditional Indigenous medicines	
21a	*[If the patient indicated they haven't used any CAM/TM, then ask:]*<br>**You indicated you haven't used any complementary, alternative or traditional Indigenous medicines; is there a reason for this?**<br>**Are there any other complementary, alternative or traditional Indigenous medicines you have used that we haven't asked you about?**
21b	*[If the patient has used CAM/TM then ask:]*<br>**Can you tell me whether you have told any of your health professionals, like your nurse, health workers, doctors or Oncologists about using CAM/TM?**<br>*Prompts: Did you decide to tell them?*<br>*Did they ask you whether you are using CAM/TM?*<br>*If you felt able to tell them, can you tell me why?*<br>*If you felt unable to tell them, can you tell me why?*<br>*Has your health professional discussed with you about future use of CAM/TM? If so, what was the outcome? (eg. Told to stop using it)*
22	**Can you tell me about your experience of using Traditional Indigenous Medicines**<br>*Prompts: What have you been using?*<br>*How often are you using it?*<br>*Where are you getting/accessing it?*<br>*Why are you using it? Any specific reasons?*

Thank you for time and for sharing your experiences with me

Semi-structured interview questions for Health Care Professionals

Semi-structured interviews will be conducted with health professionals' to explore their perspectives and experiences about Indigenous gynaecological cancer patients attending their clinics.

Interview guide

Review participant information sheet and participant and interviewer to sign consent form.

Reinforce that participation is voluntary and can be withdrawn at any time. Not all questions have to be answered.

This is your story of your experiences providing health care to people with cancer, so you will be talking about both the experiences of other people with cancer as well as your experiences.

We ask the interview to be audio recorded to make sure we accurately represent what you have to say. However, I emphasise that everything you tell me today is completely confidential. Any information that is potentially identifiable will be removed from the interview transcript, and your name will not be recorded anywhere.

**Questions**	
**Your role**	
1	Can you tell me about your work here?<br>*Prompts:*<br>- *What is your current position? Area of expertise?*<br>- *How long have you worked here?*
**Indigenous patients with cancer**	
2	Do you see many Indigenous people with cancer in your role?
3	Do you know if a patient is of Aboriginal and/or Torres Strait Islander background?
4	Can you tell me about your experiences with Aboriginal or Torres Strait Islander cancer patients?
5	What do you see as the issues facing Indigenous people with cancer?
6	From your experience, do you think that the cancer experiences of Indigenous people are different in any way to non-Indigenous people? (*Individual experience and consequences to family and broader community?*)
7	Are there any specific factors may you need to consider when treating Indigenous patients from remote locations?<br>If yes, can you describe what these are?
**Informing patients**	
8	Tell me about the type of information you provide to Aboriginal or Torres Strait Islander cancer patients about their cancer diagnosis or treatment options?
9	Is this the same information you provide to all your patients?
10	Are there particular types or modes of information or patient education that you feel work better for Indigenous patients?

11	Do you think Indigenous patients at the *[HOSPITAL NAME]* are well informed about their cancer and their treatment options? [*If not*] Why do you think this is?
**Treatment**	
12	Can you tell me about your experiences with Aboriginal or Torres Strait Islander cancer patients and their uptake/completion of treatment?
13	If patients in general don't complete their treatment, what are the reasons why they don't complete?
14	Are there any specific issues that you think are particular for Indigenous patients as to why they don't complete their treatment?
15	We know from the literature that Indigenous cancer patients are more likely to have co-morbidities than non-Indigenous patients. Do they consult with you about their co-morbidities? And do you think this influences their treatment completion?
**Complementary, alternative and traditional medicines**<br><br>Complementary and Alternative medicines (or CAM) refer to any therapies, medicines, herbal and nutritional supplements that sit outside of the dominant health system. For example, meditation, Homeopathy, Naturopathy, Traditional Chinese Medicine. Traditional Indigenous Medicines include the use of singing/chanting, bush medicine, traditional healers and external remedies prepared for healing or prevention purposes.	
16a	As part of your role, do you have any experience with patients using complementary, alternative or traditional medicines?
	IF YES, Continue<br>*IF NO, go to Question 21*
16b	Can you tell me about your experience with Indigenous patients using CAM/TM?
17	Do you ask patients if they are using any CAM/TM? Or do they bring it up first with you?
18	Do you find they are open with you about their CAM/TM use?
19	Did you discuss with them about their future use of CAM/TM? If so, did you recommend they stop or reduce using it? Did you modify the treatment you provide based on their CAM/TM use?
20	Have you ever recommended an Indigenous patient use CAM/TM before? If so, what did you recommend and why did you recommend it?
21	I want to ask you specifically about Traditional Indigenous Medicines. Are you aware of any of your Indigenous patients using Traditional Indigenous Medicines?
22	Are you aware of any of the benefits of Indigenous patients using Traditional Indigenous Medicines? If so, can you please explain these benefits?
23	Do you have any concerns around safety if your patients were using Traditional Indigenous Medicines? If so, can you please explain these concerns?
**Summary**<br>Is there anything else you would like to say about Indigenous people and cancer?	

Thank you for time and for sharing your experiences with me in this interview, your contribution to research on the care and wellbeing of cancer patients is sincerely appreciated.
